# Overview of Alzheimer's Disease and Some Therapeutic Approaches Targeting A*β* by Using Several Synthetic and Herbal Compounds

**DOI:** 10.1155/2016/7361613

**Published:** 2015-12-28

**Authors:** Sandeep Kumar Singh, Saurabh Srivastav, Amarish Kumar Yadav, Saripella Srikrishna, George Perry

**Affiliations:** ^1^Department of Biochemistry, Faculty of Science, Banaras Hindu University, Varanasi 221 005, India; ^2^Department of Biology, The University of Texas at San Antonio, San Antonio, TX 78249, USA

## Abstract

Alzheimer's disease (AD) is a complex age-related neurodegenerative disease. In this review, we carefully detail amyloid-*β* metabolism and its role in AD. We also consider the various genetic animal models used to evaluate therapeutics. Finally, we consider the role of synthetic and plant-based compounds in therapeutics.

## 1. Alzheimer's Disease (Overview)

Alzheimer's disease (AD) is the most common neurodegenerative disorder characterized by progressive memory loss. In 1907, Alois Alzheimer was the first to report a case of intellectual deterioration with the histological findings of senile plaques and neurofibrillary tangles [1]. An estimated 4.5 million Americans have AD and, as the elderly population continues to grow, the prevalence could increase by threefold to 13.2 million by 2050 [2]. The scenario seems more alarming, as it is estimated that by the year 2020, approximately 70% of the world's population aged 60 and above will be living in developing countries, with 14.2% in India. Previous reports suggest that age-adjusted prevalence of AD to be 1.91% in a community residing population in a southern Indian province of Kerala, as a part of the cognition in older adults in Trivandrum (COAT) study [3]. The reported incidence rates for AD have been lower in Asian countries than in the industrialized world [4, 5]. The impact of AD on health care costs, including direct and indirect medical and social services, is currently estimated to be greater than $100 billion per year [6]. In addition, there is currently no cure for AD; therefore, the major challenges for the near future will be the development of new therapies and therapeutic targets for disease modification and prevention.

To date, there are two major neuropathological features for the diagnosis of AD, namely, the extracellular plaque formation and neurofibrillary tangles (NFTs) formation intracellularly. The former comprises amyloid-*β* protein (A*β*) while the latter involves neurofibrillary tangles (NFTs) consisting of paired helical filaments of hyperphosphorylated tau protein. These histopathological lesions are mainly confined in the hippocampus region of the brain and in the cerebral cortex, the two large forebrain domains related to memory and other higher cognitive functions. The characteristic pathology in due course leads to the typical clinical symptoms, for example memory impairment, general cognitive decline, and personality changes associated with AD. The causes of AD are still rather poorly known, with different etiologies (e.g., A*β* overproduction, genetics, A*β* impaired clearance, and NFT formation) leading to senile plaques, neurofibrillary tangle (NFT) formation, and extensive neuronal death. However, several studies and evidence point to A*β* as critical in the pathogenesis of AD. According to the amyloid cascade hypothesis, A*β* peptides form aggregates and toxic assemblies which initiate several processes leading to neuronal dysfunction and ultimately large-scale cell death [7]. The prevalence of AD varies among several different factors, including age, genetics, comorbidities, and education level. There is no way to absolutely diagnose AD without performing an autopsy. There is no cure for AD; however promising research and development for early detection and treatment is underway.

### 1.1. History

Alzheimer's disease was discovered by a German Neurobiologist and Psychiatrist named Alois Alzheimer in 1906 [9]. Before Alzheimer's 1906 discovery, both scientists and the nonscience community viewed dementia as a “natural” progression of age, and “senility was accepted as a part of aging” according to Natalie Whaley in her honours thesis on the social history of Alzheimer's disease. First time, AD was observed in Auguste D., a 51-year-old woman. Her family observed some unusual behavioural changes in her personality and then they brought her to Dr. Alois Alzheimer in 1901. The family reported problems with memory loss, speaking difficulty, loss of good judgement, disorientation to time and place, and problem with abstract thinking. Later on, Dr. Alzheimer described that she is having an aggressive form of dementia, memory impairment, problem using language, and behavioural changes [10]. Dr. Alzheimer also noted many other abnormal symptoms, including rapid mood swing, personality changes, loss of initiative, sleeping longer than usual, and loss of interest in usual activity [11]. Dr. Alois followed her for about five years, until her death, in 1906. After death, he performed an autopsy and found dramatic shrinkage of the cerebral cortex, deposition of fat bodies in blood vessels, and atrophied brain cells [9]. He discovered neurofibrillary tangles and senile plaques, which have become indicative of AD [11]. The condition was first discussed in the medical literature in 1907 and named after Alzheimer in 1910.

### 1.2. Pathology and Pathophysiology of Alzheimer's Disease

The brain of AD patient often shows marked atrophy, with broadened sulci and shrinkage of the gyri. In the majority of cases, every part of the cerebral cortex is involved. However, the occipital lobe is often relatively spared. The cortical ribbon may be thinned and ventricular dilatation is apparent, especially in the temporal horn, due to atrophy of the amygdala and hippocampus. During the last couple of decades, the pathology of AD has been extensively studied whereby animal models have provided valuable information in understanding of the pathogenic mechanisms of AD. Furthermore, AD pathology can be divided into three broad sections: (a) positive lesions (lesions related to accumulation), (b) negative lesions (those that are due to losses), and (c) inflammation and plasticity (those that are due to the reactive processes). The first category involving positive lesions is very common and easy to detect and constitutes the basis of the diagnosis. Both the neuronal and synapse loss are difficult to evaluate, as they do not belong to the diagnostic criteria but could be the alterations that are more directly related to the cognitive deficit. Furthermore, microscopic studies on AD brain revealed significant neuronal loss, in addition to shrinkage of large cortical neurons. Many neuropathologists believe that loss of synapses neurons in association with shrinkage of the dendritic arbor of large neurons is the critical pathological substrate. The main neuropathological hallmarks of AD are senile plaques and neurofibrillary tangles, although these two are not unique to AD and can be found in other human neurodegenerative disorders and in clinically normal individuals as well. Apart from senile plaques, two other types of amyloid-related plaques are found in the brains of AD patients: burnt-out plaques, which consist of an isolated dense amyloid core and diffuse plaques, which contain poorly defined amyloid but no well-circumscribed amyloid core. It is believed that the abnormal processing of the amyloid-*β* protein precursor through amyloidogenic pathway results in different fragments, the most toxic of which is the A*β*_42_ peptide [12]. A*β*_42_ readily self-aggregates and forms clumps of insoluble fibrils in the brain, thereby triggering the formation of senile plaques. It has been postulated that A*β*_42_ is mainly responsible for initiating a cascade of events leading to neuronal dysfunction, later followed by death. Although increasing evidence supports the hypothesis that the accumulation of A*β* is very decisive to the pathogenesis of AD [13], some investigators believe that A*β* is not exclusively responsible for the neuronal alterations that underlie its symptoms [14].

Neurofibrillary tangles (NFTs) are the other important characteristic histopathological features of AD. Neurofibrillary tangles are found inside neurons and are composed of paired helical filaments of hyperphosphorylated microtubule-associated protein tau (MAPT). Accumulation of NFTs intracellularly may cause dysfunction of the normal cytoskeletal architecture of neurons with subsequent death. Senile plaques (A*β*) and neurofibrillary tangles (NFTs) are not distributed evenly across the brain in AD but are confined to vulnerable neural systems.

Other pathological modifications commonly discovered in the brains of AD patients include granulovacuolar degeneration, neuropil threads, and amyloid angiopathy. The latter one is a distinct vascular lesion and found in many AD brains, consisting of amyloid deposition in the walls of small- to medium-sized cortical and leptomeningeal arteries due to which the involved vessels may become compromised with resultant hemorrhage.

After microscopic examination, observation of sufficient amount of senile plaques and neurofibrillary tangles suggests important pathological criteria for the diagnosis of AD. Because of the presence of amyloid-*β* in senile plaques and to a variable degree in cerebral blood vessels in the AD brain, the roles of this important protein and its precursor peptide, amyloid-*β* protein precursor, have been widely investigated [15], although the exact nature of their roles in the pathogenesis of AD remains unclear. Increasingly, the importance of differential neuronal vulnerability and the relationship of this to the morphological and biochemical characteristics of AD are being recognized. The most consistent neurochemical change associated with AD has been the well-documented decline in cholinergic activity that has inspired many attempts to treat AD with cholinergic drugs. However, additional deficiencies in glutamate, norepinephrine, serotonin, somatostatin, and corticotrophin-releasing factors have also been described.

### 1.3. Risk Factors for AD

**Figure 1:**
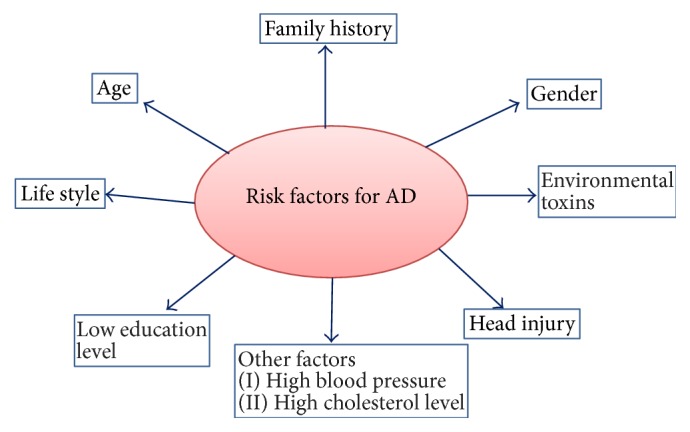
Showing different causal and risk factors for Alzheimer's disease.

While scientists know that distortion of nerve cells in case of Alzheimer's disease occurs, why this happens is still unknown. However, they have discovered certain risk factors that increase the likelihood of developing Alzheimer's (Figure 1).

#### 1.3.1. Age

With increasing age, the risk of developing AD becomes higher. Most patients develop AD after the age of 65 years. The risk of developing AD reaches 50% for individuals above the age of 85 years. Statistically speaking, about 5% of men and women between the ages of 65 and 74 have Alzheimer's disease, and nearly half of those aged 85 and older may have the disease. Despite its prevalence, Alzheimer's disease is not a normal part of aging. The age-specific incidence rates for Alzheimer's disease demonstrate a doubling of incidence for about every six years of added life, which indicates an exponential increasing risk with increasing age of individuals. This exponential risk is somewhat similar across studies, regardless of geographic region, even if the underlying absolute incidence rate differs.

#### 1.3.2. Familial History and Genetics

Another risk factor involves family history. Researchers have shown that those who have a parent, brother, or sister with AD are more likely to develop the disease than individuals who do not have a first-degree relative with AD. The vast majority of AD cases are not genetically inherited, although some genes may act as risk factors [16]. The risk increases if more than one family member have the illness. Genetically identified forms of AD, which usually have an onset before the age of 65, have been identified and account for 0.1% of disease cases [17]. Scientists have identified three genes that described people who will develop Alzheimer's, but only a very small percentage of individuals with AD (about 1%) carry these genes. The apolipoprotein E (APOE-*ε*4) is carried by about 25% of individuals and increases the risk of developing AD, but it is not sure that individuals with APOE-E4 will develop the disease. Scientists believe that the vast majority of AD cases are caused by a complex combination of genetic and nongenetic determinants.

#### 1.3.3. Other Risk Factors

Besides age, family history, and genetics, other important risk factors exist that may contribute to AD risk. In this context, some promising research suggests that strategies for keeping and living overall healthy aging may help maintain brain health and may even provide some protection against AD. These factors include eating habits, healthy lifestyle, staying socially and physically active, and avoiding excess alcohol and tobacco.

Some of the strongest evidence links brain health to heart health. The risk of developing Alzheimer's disease or vascular dementia appears to be increased by many conditions that damage the heart and blood vessels. These include heart disease, diabetes, stroke, high blood pressure, and high cholesterol. It is a common saying in medical practice that “work with your doctor to monitor your heart health and treat any problems that arise.” Further, studies of donated brain tissue provide additional evidence for the heart-head connection. These studies suggest that plaques and tangles are more likely to cause Alzheimer's symptoms if strokes or damage to the brain's blood vessels is also present.

## 2. Different Hypotheses Postulated Related to Alzheimer's Disease

### 2.1. The Tau Hypothesis of Alzheimer's Disease

Tau protein plays a critical role in pathophysiology of AD. The tau hypothesis focuses primarily on the role of the microtubule binding tau protein, which is the main component of NFTs in AD. Hyperphosphorylation of tau protein results in NFT formation. This hypothesis proposes a mechanism for neurotoxicity based on the loss of microtubule-stabilizing tau protein that leads to degradation of the cytoskeleton [18]. However, it is not clear whether tau hyperphosphorylation is responsible or is caused by the formation of abnormal helical filaments [19]. Tauopathy-like diseases also support the tau hypothesis in which the same protein is significantly misfolded [20]. However, a majority of research groups support the alternative hypothesis that amyloid-*β* is the primary causative agent for AD [19].

### 2.2. The Cholinergic Hypothesis of Alzheimer's Disease

The cholinergic hypothesis is the oldest AD hypothesis [21]. This hypothesis proposed that AD is caused by the reduced synthesis of a neurotransmitter called acetylcholine in neurons. The cholinergic hypothesis was formulated over 30 years ago and suggests that a dysfunction of acetylcholine-containing neurons in the basal forebrain contributes substantially to the cognitive decline observed in AD patients [22]. In addition to the dysfunction and neuronal loss in basal forebrain regions, confirmation of cholinergic losses comes from studies that report deterioration in the activity of acetylcholine esterase (AChE) and choline acetyltransferase (ChAT), reduced acetylcholine (ACh) release, and decreased level of nicotinic and muscarinic receptors in the AD affected brain [23]. This observation led to the formation of the cholinergic hypothesis, considered the oldest hypothesis of AD [24, 25]. The cholinergic hypothesis has not had widespread support, largely because medications intended to treat acetylcholine deficiency have not been very effective, although 4 of the 5 approved drugs work on just this mechanism. Other cholinergic effects have also been proposed, for example, initiation of large-scale aggregation of amyloid leading to generalized neuroinflammation [26].

### 2.3. The Mitochondrial Cascade Hypothesis

The mitochondrial cascade hypothesis was first proposed by Swerdlow and Khan, 2004, which postulates that mitochondrial dysfunction is the primary cause of A*β* deposition, neurofibrillary tangle (NFT) formation, and synaptic degeneration in AD [27]. The mitochondrial cascade hypothesis takes several conceptual liberties. It assumes that similar physiologic mechanisms underlie AD and brain aging. It postulates that because AD mitochondrial dysfunction is systemic, it cannot simply represent a consequence of neurodegeneration. The mitochondrial cascade hypothesis argues that non-Mendelian genetic factors contribute to nonautosomal dominant AD. Finally, it posits that AD brain mitochondrial dysfunction drives to amyloidosis, tau phosphorylation, and cell cycle reentry. Mitochondrial dysfunction is observed in several AD tissues [28], including platelets, fibroblast, mitochondria, and brain. There are basically three mitochondrial enzymes that are found to be defective. This includes reduced activities of *α*-ketoglutarate dehydrogenase complex, cytochrome oxidase, and pyruvate dehydrogenase complex [29]. Through special analysis of AD brains, level of cytochrome oxidase is found to be normal, but the enzyme itself is structurally altered [30]. In AD, oxidative stress and proteasome dysfunction have been postulated to facilitate mitochondrial dysfunction [31]. Also, studies on cytoplasmic hybrid (cybrid) indicate that mtDNA at least, in part, accounts for reduced cytochrome oxidase activity in AD [32].

### 2.4. Amyloid Cascade Hypothesis

Although the exact cause of AD is still a matter of debate, the amyloid cascade hypothesis is the best accepted and most studied hypothesis among those mentioned above. The presence of amyloid plaques is considered to be the main characteristic of AD pathology. The primary constituent of senile plaques identified so far is A*β* peptide, which is produced on account of proteolytic processing of the amyloid-*β* protein precursor (A*β*PP) by *β*- and *γ*-secretases [33, 34]. Furthermore, cloning of the A*β*PP gene [35] has allowed the disease to be examined at molecular and biochemical levels. Subsequently, mapping of several familial forms of AD (fAD) mutations in the A*β*PP gene [36], the association of AD with Down's syndrome, and higher prevalence of AD with increased numbers of A*β*PP all established the critical role of A*β*PP in AD pathogenesis [33, 37, 38]. The central role of A*β*PP in AD etiology is further supported by the identification of fAD mutations in presenilin 1 (PS1), which involves A*β*PP cleavage and generates A*β* and AICD fragments.

**Figure 2:**
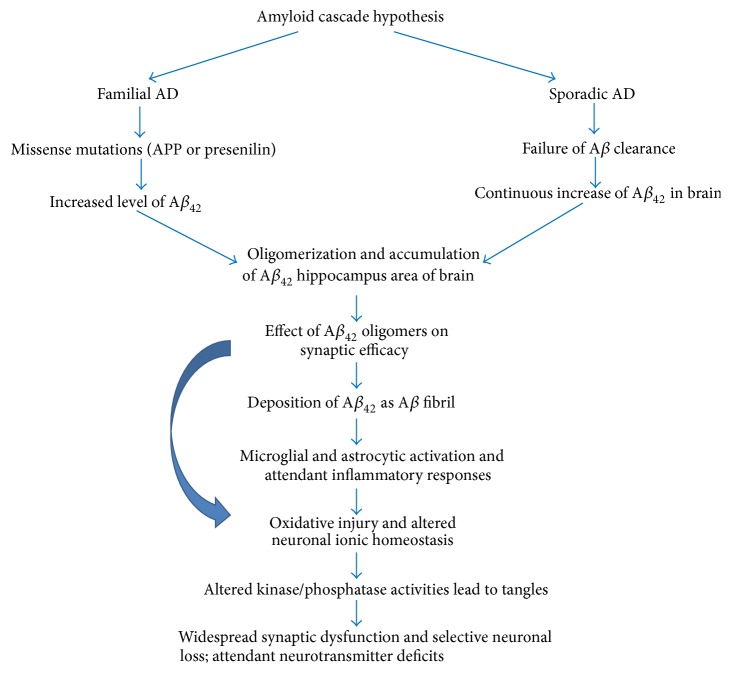
The amyloid cascade hypothesis of Alzheimer's disease. This hypothesis represents the classic theory of the origins of AD. Both familial forms of Alzheimer's (fAD) and later-onset forms with no known etiology (sporadic AD) lead to the production of excess A*β*_42_. Once this toxic peptide begins to aggregate, a cascade of events is triggered that produces the biological and neurological symptoms of Alzheimer's disease.

In the early 1990s, it was proposed that the main essence of the amyloid cascade hypothesis is increased production or decreased clearance of A*β* peptide, the culprit behind AD [39, 40]. Accumulation of the hydrophobic A*β* peptide (A*β*_40_ and A*β*_42_) results in its self-aggregation and formation of insoluble plaques, triggering a cascade of events resulting in death of the neuronal cells and thus causing AD (Figure 2).

A large portion of fAD cases is accounted for by mutations in the presenilin 1 (PS1) gene [41]. PS1 is one of the four main membrane proteins in presenilin complex which associates with three other membrane proteins to form the *γ*-secretase complex. Unlike in A*β*PP, fAD mutations in PS1 are scattered throughout the length of molecules. Many of these mutations result in modified cleavage of A*β*PP, causing enhanced production of the longer A*β*_42_ peptide, which is more prone to self-aggregate as compared to the smaller A*β*_40_ [42] and is shown to be more toxic* in vitro*. Since the level of A*β*_42_ is found to be much higher in AD patients, it was postulated that the rise in levels of A*β*_42_ triggered the cascade of the distorting events resulting in AD [43]. Although there is increase in A*β*_42_ due to many fAD mutations, some mutations in PS1 do not elevate A*β*_42_ levels but rather decrease A*β*_40_ levels. This has led to yet another possibility that an enhancement in the A*β*_42/40_ ratio, instead of the absolute levels of A*β*_42_, is pathogenic and triggers the deleterious events leading to the disease. This view is supported by the observation that increased A*β*_42/40_ ratio is generally inversely related to the age of onset of AD [44].

## 3. Amyloid-*β* Protein Precursor (A*β*PP) and Its Function

The amyloid-*β* protein precursor (A*β*PP) gene is located on chromosome 21 in humans. Alternative splicing of the A*β*PP transcript generates 8 isoforms, of which 3 are most common: A*β*PP695, A*β*PP751, and A*β*PP770. The 695 amino acid form predominantly expresses in the CNS, and 751 and 770 amino acid forms express ubiquitously [45]. A*β*PP belongs to type I transmembrane proteins that include the amyloid precursor-like proteins (APLP1 and APLP2) in mammals and the amyloid precursor protein-like (APPL) in* Drosophila*.

The exact physiological function of A*β*PP is not well known and remains an important issue in AD research. In many studies, overexpression of A*β*PP shows a positive effect on cell health and growth. This effect is epitomized in transgenic mice that overexpress wild-type A*β*PP and have enlarged neurons [46]. A*β*PP knockout mice are viable and fertile, showing a comparatively subtle abnormal phenotype [47, 48]. APLP1 and APLP2 knockout mice also survive and are fertile. However, double null mice A*β*PP/APLP2 and APLP1/APLP2 and triple null mice A*β*PP/APLP1/APLP2 show early postnatal lethality [49, 50]. Interestingly, A*β*PP/APLP1 mice are viable [50], suggesting that APLP2 is critical when either A*β*PP or APLP1 is absent.

Further, the similarity in proteolytic processing and topology between Notch and A*β*PP suggest that A*β*PP may function as a membrane receptor like Notch. Indeed, different A*β*PP ligands have been identified, such as A*β* [51], netrin-1 [52], and F-spondin [53]. However, while binding of A*β*PP by these ligands can affect A*β*PP processing, the exact downstream signalling events triggered by such binding remain to be clarified and an authentic membrane receptor function for APP remains speculative.

Although A*β*PP has been the subject of much study since its identification, its physiological function remains unclear. A*β*PP has important roles in neurites' outgrowth and synaptogenesis, cell adhesion, calcium metabolism, neuronal protein trafficking along the axon, and transmembrane signal transduction, among others, all requiring additional* in vivo* experimental evidence [54]. A*β*PP generates various fragments during proteolytic processing and these A*β*PP metabolites serve various functions. Therefore, the net effect of full-length A*β*PP on biological activity may be a combination of its metabolites' functions, depending on the proportion of levels of each A*β*PP metabolite. In adult animals, intracerebral injections of the A*β*PP ectodomain can improve cognitive function and synaptic density [55, 56]. The sites most responsible for the bioactivity of the A*β*PP ectodomain appear to be its two heparin-binding domains [57]. Overall, studies from various research groups suggest that A*β*PP plays an important role in protein trafficking regulation.

## 4. Proteolytic Processing of A*β*PP and Generation of A*β* Peptide

As described above, A*β*PP is a type I transmembrane protein. It is synthesized in the endoplasmic reticulum (ER) and transported to the trans-Golgi-network (TGN) through the Golgi apparatus where the highest concentration of A*β*PP is found in neurons at steady state [58–60]. One of the most prominent areas of AD research is the study of the generation of A*β* after A*β*PP processing. A*β* generation takes place in ER and Golgi/TGN [60]. Further, A*β*PP can be transported from the TGN to TGN-derived secretory vesicles to the cell surface where it is either reinternalized via an endosomal/lysosomal degradation pathway [61, 62] or cleaved by *α*-secretase to produce a soluble molecule, A*β*PPs_*α*_ [63]. Some reports also suggest involvement of endosomal/lysosomal system in A*β* generation [64]. Unlike A*β* which is neurotoxic, studies suggest that A*β*PPs_*α*_ is neuroprotective, making the subcellular distribution of A*β*PP an important factor in neurodegeneration [65]. Therefore the characterization of the mechanisms involved in APP transport and trafficking are crucial to understanding the pathogenesis of AD.

**Figure 3:**
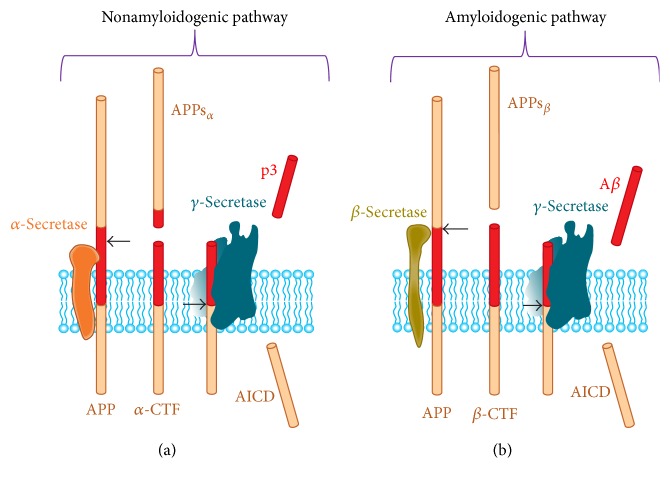
Processing of the amyloid-*β* protein precursor (A*β*PP) occurs by two pathways. (a) Nonamyloidogenic processing of A*β*PP involving action of *α*-secretase followed by *γ*-secretase as shown in the figure. (b) Amyloidogenic processing of A*β*PP involving *β*-secretase followed by the action of *γ*-secretase. Both processes generate soluble ectodomains (A*β*PPs_*α*_ and A*β*PPs_*β*_) and a similar intracellular C-terminal fragment (AICD). The A*β* peptide starts within the ectodomain and continues into the transmembrane region (red). Adapted from Thinakaran and Koo [8].

Processing of A*β*PP takes place by two different pathways: amyloidogenic pathway, which results in the generation of toxic A*β* fragment of 42 amino acid, and nonamyloidogenic pathway, which is required for normal functioning of neurons. There is involvement of three different types of serine proteases in the A*β*PP processing which are *α*-, *β*-, and *γ*-secretases. The quantitatively and functionally most important proteolytic processing of A*β*PP is mediated through nonamyloidogenic pathway, that is, cleavage of A*β*PP by the action of *α*- and *γ*-secretases. Action of *α*-secretase releases the A*β*PPs_*α*_ ectodomain and a carboxy terminal fragment (A*β*PP-CTF_*α*_). Action of *γ*-secretase later generates a small p3 and AICD fragment (Figure 3). A*β*PPs_*α*_ has been suggested to show neuroprotective and synapse-promoting activities [66], but the mechanism behind this and identification of the receptor mediating these effects has not yet been identified.

Cleavage of A*β*PP by the action of *β*-secretase and *γ*-secretase results in A*β*_42_ generation, called amyloidogenic pathway (Figure 3). Action of *β*-secretase releases the ectodomain A*β*PPs_*β*_ and rests A*β*PP carboxy-terminal fragment (A*β*PP-CTF_*β*_) which is further cleaved by the *γ*-secretase producing the A*β* peptide(s) and the A*β*PP intracellular domain (AICD) fragment. The biological function of all the above fragments (A*β*PPs_*β*_, A*β*, and AICD) generated through amyloidogenic pathways is still to be explored, although A*β* release is associated with synaptic activity and synaptic transmission onto neurons [67]. The AICD fragment is thought to be a nuclear signalling molecule [68], but this is also not fully explored [69].

## 5. Definition of Amyloid-*β* Peptide

Definition of amyloid-*β* follows the guidelines of nomenclature established in the November 2006 meeting of The Nomenclature Committee of the International Society of Amyloidosis. Amyloid-*β* is defined as peptides of 36–43 amino acids that are primarily involved in AD as the main component of the amyloid plaques found in the brains of AD patients. Amyloid-*β*, also defined as protein deposits found* in vivo*, can be distinguished from nonamyloid protein deposits by observing under an electron microscope. Amyloid-*β* has a characteristic fibril appearance, a unique pattern of X-ray diffraction, and an affinity for the dye Congo red of histological samples, which results in an apple green birefringence under plane-polarized light [70]. The term amyloid-*β* was initially reported to restrict as extracellular deposits only. However, many types of amyloid-*β* have since been reported to begin intracellularly, resulting in the characteristic extracellular amyloid deposits found upon cell death. Therefore, amyloid is no longer restricted to extracellular inclusion but also includes those intracellular inclusions having typical amyloid appearance [70].

## 6. Physiological Function of A*β* Peptide

Multiple lines of evidence reveal that overproduction of A*β* through A*β*PP processing results in a neurodegenerative cascade leading to self-aggregation, synaptic dysfunction, formation of intraneuronal fibrillary tangles, and gradual neuron loss in the hippocampus [71]. There are two main toxic species, A*β*_40_ and A*β*_42_, with A*β*_42_ being more hydrophobic in nature and more prone to self-aggregation which results in A*β* fibril formation [72]. Previous studies on familial form of AD (fAD) mutations consistently show rise in the ratio of A*β*_42/40_ [73], thereby indicating that elevated levels of A*β*_42_ relative to A*β*_40_ are crucial for AD pathogenesis, likely achieved by providing the core for A*β* assembly into oligomers, fibrils, and amyloidogenic plaques [74]. Although the majority of A*β* peptides are secreted from the cell, A*β* can be generated in several subcellular compartments within the cell, such as the ER, Golgi/TGN, and endosome/lysosome. In addition, internalization of extracellular A*β* can be done by a cell for its degradation. The presence of intracellular A*β* implies that A*β* may accumulate within the neuronal cell and contribute to AD pathogenesis. Confirming the presence of intracellular A*β*, intraneuronal A*β* immunoreactivity has been found in the hippocampal and entorhinal cortical regions which are more prone to early AD pathology with mild cognitive impairment (MCI) in AD patients [75]. The accumulation of intracellular A*β* paves the way for extracellular plaque formation in Down's syndrome (DS) patients [76] and the level of intraneuronal A*β* reduced as the extracellular A*β* plaques accumulate [77]. Studies of transgenic mouse models consistently confirm evidence for intracellular accumulation of A*β* as an early event in the neuropathological phenotype, with decreasing intraneuronal levels of A*β* as extracellular plaques build up [78–80]. Intraneuronal A*β* can also disrupt amygdala-dependent emotional responses by modulating the ERK/MAPK signalling pathway [81]. Previous reports also suggest reduction in A*β* neurotoxicity due to inhibition of dynamin-mediated but not clathrin-mediated A*β* internalization [82]. One recent study by Friedrich et al. suggests that intracellular A*β* can self-aggregate within the cell and disrupt the vesicular membrane, contributing to its pathological effect [83].

A*β* was originally considered a neurotoxic species confined to the brain of aged or demented persons. Later findings suggest that the presence of soluble A*β* species in the bodily fluids of many species [84] and in the conditional cell culture media [85] has disproved this concept and inferred a physiological function for A*β*. Low levels of A*β* enhance hippocampal long-term potentiation and improve memory, indicating its novel positive, modulatory role in neurotransmission and memory [86], while excessive A*β* causes neuronal loss as well as synaptic dysfunction. One study using a transgenic* Caenorhabditis elegans* model found that intracellular A*β* aggregation in muscle cells may trap excess free copper to reduce copper-mediated cytotoxic effects [87]. However, whether A*β* can form intracellular aggregates in human peripheral cells to exert a physiologically protective function remains to be determined.

## 7. Mechanism of Formation of A*β* Fibril

**Figure 4:**
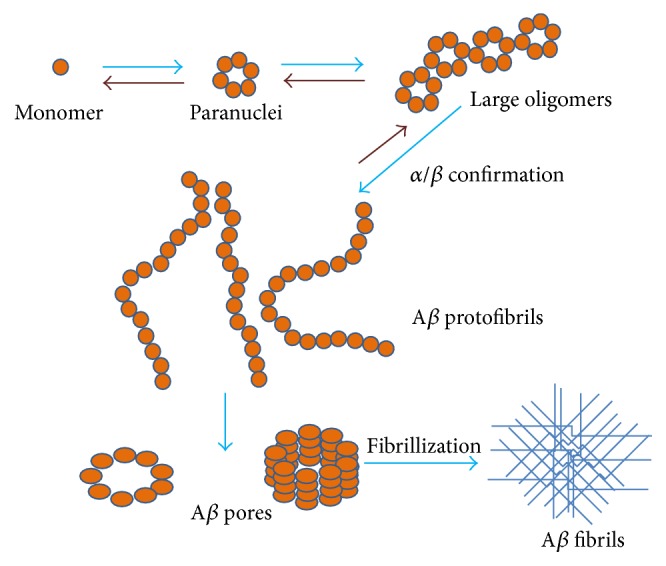
A model showing A*β*_42_ oligomerization and fibrillization: the equilibrium between monomer to paranuclei and from paranuclei to large oligomers is rapid and reversible. The conversion of oligomers to protofibrils is slower but also reversible. Conversion of protofibrils into fibrils is an irreversible step. Basically, the monomers, paranuclei, and large oligomers do not have any definite structure instead of some *β*-turn/*β*-sheet and helical (*α*) elements. Essential conformational changes occur during protofibril formation where the unstructured, *α*-helix, and *β*-strand elements transform into *β*-sheet/*β*-turn structures.

The mechanism of formation of A*β* oligomer* in vivo* remains unclear. In this context, Glabe suggests that the complexity of the oligomer formation can be assumed by the fact that multiple A*β* oligomer conformations are produced via different pathways [88]. The mechanisms of fibril formation of extracellular and intracellular oligomers may also vary. The fibrillization of A*β* into senile plaques is a complex process involving several steps [89–91]. After A*β* is released from cells, it can bind to several proteins: for example, albumin, *α*1-antichymotrypsin, apolipoprotein E, and complement proteins [92]. Presence of A*β* as stable soluble dimers is detected in both cell culture media and brain homogenates [93]. Total A*β* concentration may be the critical determinant of fibril formation. In normal brain, breakdown of A*β* takes place immediately after its production from the cells before fibrillization or deposition, while, in the aging brain, increased production of A*β* and its reduced rate of clearance may lead to A*β* fibrillization, further leading to disease condition and AD pathogenesis. Recent studies reveal three different types of A*β* oligomers: (a) very short oligomers ranging from dimer to hexamer size [94, 95]; (b) small oligomers ranging from 17 to 42 kDa which are A*β*-derived diffusible ligands (ADDLs) [96]; and (c) protofibrils that can be seen in electron microscopy as short fibril intermediates of less than 8 nm in diameter and less than 150 nm in length. Protofibrils are short-lived structures detected during* in vitro* formation of mature amyloid fibrils [97–99]. However, relationships between the aforementioned oligomers remain unclear. Moreover, all oligomeric forms of A*β* derived intermediates, that is, oligomers, ADDLs, protofibrils, and mature A*β* fibrils, are potentially neurotoxic and may be a key cause of neurotoxicity in AD. A*β* exists mainly in two alloforms: A*β*_40_ and A*β*_42_, which follow distinct oligomerization pathways [94, 100]. Each peptide showed different behaviour at the earliest stage of assembly and monomer oligomerization. Kinetic studies of A*β* fibril formation have shown that formation of A*β*_42_ self-aggregates is faster than A*β*_40_ and forms fibril [98, 101]. It is also well reported that the fibrillogenic and neurotoxic property of A*β*_42_ is higher than that of A*β*_40_. The initial phase of fibrillization of A*β*_42_ monomers involves formation of pentamer/hexamer units called paranuclei (Figure 4). These paranuclei are initial structures that can further oligomerize to larger units and form large oligomers, protofibrils, and fibrils. Monomers, paranuclei, and large oligomers are predominately unstructured with only short *β*-sheet/*β*-turn and helical elements. During formation of protofibrils, essential conformational changes take place when the unstructured, *α*-helix, and *β*-strand elements convert into *β*-sheet/*β*-turn structures. Paranuclei could not be detected for A*β*_40_ at similar concentrations of the peptide. Aggregate-free A*β*_40_, when carefully prepared, existed as monomers, dimers, trimers, and tetramers, in rapid equilibrium [94]. The important residue promoting the pentamer/hexamer formation is Ile41. Addition of later residue to A*β*_40_ is sufficient to induce paranuclei formation [94]. A natural propensity to form paranuclei is the only feature of A*β*_42_. This important finding may explain the predominantly strong association of A*β*_42_ with AD. Paranuclei formation in A*β*_42_ is blocked by oxidation of Met35 and produces oligomers indistinguishable in morphology and size and from those produced by A*β*_40_ [95]. Preventing the fibrillization of toxic A*β*_42_ paranuclei through selective Met35 oxidation thus represents a potential therapeutic target for AD treatment. The most important feature of controlling early oligomerization of A*β* is the length of the C-terminal as compared to 34 physiological relevant alloforms of A*β* [95]. The primary amino acid residue in A*β*_42_ is a side chain of residue 41 which is crucial for effective paranuclei formation and self-aggregation into oligomer formation. A*β*_40_ self-aggregation is particularly critical to substitutions of Glu22 or Asp23 and to truncation of the N terminus [94]. Whereas A*β*_42_ oligomerization is largely unaffected by substitutions at positions 22 or 23 or by N-terminal truncations, it is significantly affected by Phe19 or Ala21 substitutions. The above statement reveals that A*β* oligomerization differs between A*β*_40_ and A*β*_42_ which are controlled by specific regions and residues.

## 8. A*β* Toxicity

Alzheimer's disease is considered by some researchers to be a disease of the synapses and has been termed “synaptic failure” [102]. While A*β* can destroy neurons, synaptotoxicity may be more appropriate for earlier stages of AD that are best categorized by synaptic loss rather than neuronal death. Loss of dendritic spines or synaptic terminals may cause the associated deterioration in cognitive functions that characterizes AD. However, it is still unclear whether the synaptotoxic and neurotoxic actions of A*β* are a separate mechanistic process or if the actions follow a common mechanism [103]. As discussed above, the pentameric and hexameric oligomers may be the building blocks of the more toxic decameric and dodecameric complexes. Both cross-linked oligomeric forms of A*β* and A*β* fibril were significantly more toxic than disaggregated A*β* (dimers threefold and tetramers 13-fold more toxic than monomers). One of the main results is the fact that monomers have very low toxicity, while toxicity rises substantially only when A*β* self-associates, although it is still challenging to establish a degree of increasing toxicity with the number of monomers in the given oligomer because of the decrease in occurrence frequency of higher order oligomers. Monomeric A*β* has the propensity to adopt different conformations in water solutions, including momentarily extended *β*-sheet conformations in the central and C-terminal regions, connected by turn between them, or *β*-hairpin [104].

Several other lines of study support a role for oligomeric form of A*β* as the toxic entity in AD patients. Human brain shows soluble oligomers with similar structural properties as observed* in vitro* by antioligomeric antibodies staining; the same oligomers were also observed* in vivo* [105]. A*β* oligomer toxicity* in vitro* has been attributed to several distinct mechanisms, including but not limited to membrane disruption and direct formation of ion channels. There have been numerous reports of increased membrane conductance or leakage in the presence of A*β* oligomers ranging from small globulomers to large prefibrillar assemblies [106, 107], with some evidence presented to support formation of discrete ion channels of pores [108, 109].

Fibrillar A*β*, on the other hand, has been shown to bind to a wide array of cell surface proteins, including the receptor for advanced glycation end products (RAGE) complex and A*β*PP [110], leading in some cases to increased free radical formation and oxidative stress. Similarly, binding to the *α*-7 nicotinic receptor can mediate N-methyl-D-aspartate (NMDA) receptor activity with broad effects on cellular metabolism [111]. Any or all of these effects may play a role in loss of synaptic function, leading to symptomatic AD. Other proposed interactions, such as dysregulation of calcium channels, may be confounded by membrane disruption effects, making them harder to confirm.

It is important to note that since A*β* exists* in vitro* and* in vivo* as a continuum of different oligomeric states, none of which are particularly stable, it is difficult to distinguish biological effects induced by one specific type of nonfibrillar oligomer. Therefore, it is entirely feasible that A*β* has significantly different physiological effects when in different oligomeric forms. Thus, it is difficult to exclude any of the putative mechanisms for involvement of A*β* oligomers in progression of AD without further study.

**Figure 5:**
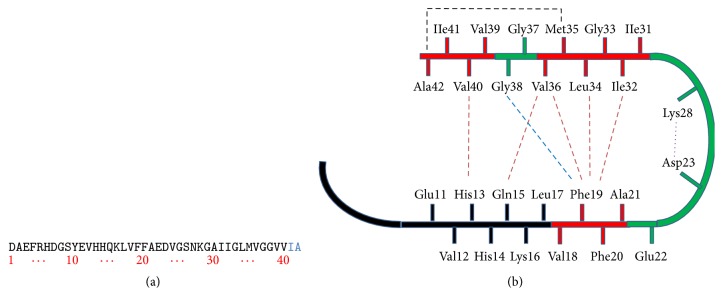
(a) Sequence of A*β*_42_ that is derived from human A*β*PP. (b) Structural constraints in A*β*_40_ and A*β*_42_ fibrils. NMR measurements of A*β*_40_ fibrils have shown that residues 1–10 are unstructured and residues 11–40 adopt a *β*-turn-*β* fold. Side chain packing is observed between Phe19 and Ile32, Leu34 and Val36, Gln15 and Val36, and His13 and Val40 (orange dashed line). In A*β*_42_ fibrils, residues 1–17 may be unstructured (in black), with residues 18–42 forming a *β*-turn-*β* fold. Molecular contacts have been reported within the monomer unit of A*β*_42_ fibrils between Phe19 and Gly38 (blue dashed line) and between Met35 and Ala42 (black dashed line). In both A*β*_40_ and A*β*_42_, the turn conformation is stabilized by hydrophobic interactions (red residues) and by a salt bridge between Asp23 and Lys28 (purple dashed line).

A*β*_40_ is the common, more soluble form of A*β*. A*β*_42_ has two extra amino acids on the end of the peptide (Figure 5(a)). One of these, 42nd amino acid, is an alanine, which can loop back to form a salt bridge with 35th amino acid, methionine (Met35) (Figure 5(b)). This extra hairpin turn of A*β*_42_ makes it less soluble and more toxic. The toxicity of A*β*_42_ is much greater than A*β*_40_.

## 9. Role of A*β* in AD Pathogenesis

As discussed so far, it is clear that A*β* is one of the hallmarks for Alzheimer's disease. A*β* is generated from A*β*PP processing by *β*- and *γ*-secretases through the amyloid cascade pathway. A*β* is one of the main toxic peptides which has a critical role in AD pathogenesis. A*β* normally has a propensity for self-aggregation, resulting in A*β* fibril formation which ultimately form senile plaques extracellularly, causing neuronal damage and synaptic dysfunction. Although A*β* aggregates are mainly found in the hippocampus area of postmortem brain of AD patients, they are also distributed to some extent in the cortex area of brain. As we know, the hippocampus is the prime memory storage part of the brain, so these aggregates affect the surrounding neurons in hippocampus area and are responsible for AD pathology. The temporal profile of pathological features, together with genetic risk factors for AD, has led to the hypothesis that accumulation of A*β* oligomers during early, preclinical stages of the disease initiates a cascade of events resulting in synaptic dysfunction, neuronal loss, and atrophy within the temporoparietal and hippocampal regions. This neurodegeneration, in turn, causes neuronal dysfunction, cognitive decline, and ultimately complete loss of memory [112].

## 10. Therapeutic Approaches for AD

**Figure 6:**
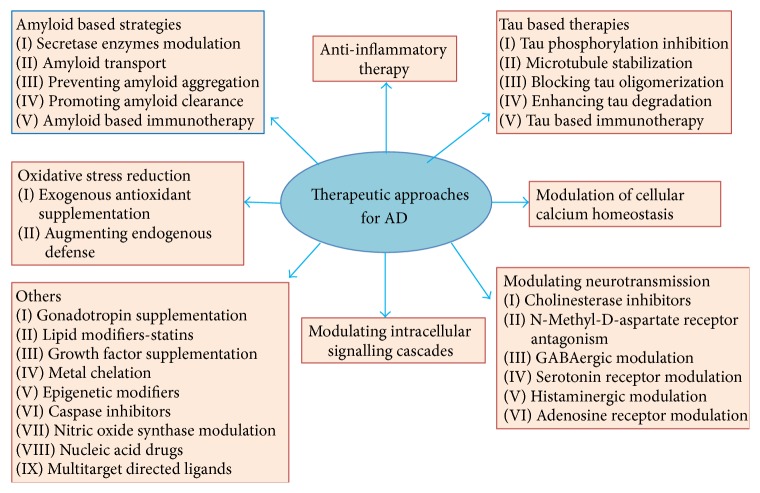
Different therapeutic approaches for the treatment of AD.

There is no cure for AD; however, drug treatments are available to help relieve symptoms in several aspects of the disease, and researchers around the world are focusing on finding better treatments, preventive strategies, and ultimately a cure. A variety of cellular mechanisms can lead to the generation of Alzheimer's disease. Along with A*β*, microtubule-associated protein tau is another hallmark of AD [113]. In the case of AD, tau becomes hyperphosphorylated, aggregated, and finally accumulated as NFT [114]. Tau plays an important role, not only as axonal protein but also as regulator of dendritic function, particularly mediating early A*β* toxicity during AD [115]. Therefore, A*β* and tau became targets in drug development for AD. Many clinical trials targeting these two proteins have been implemented; several lines of research are still under investigations. There are several therapeutic approaches being investigated for the treatment of AD (Figure 6). Therapeutic strategies are basically categorized in the following three ways: (i) treatments that prevent the onset of the disease by sequestering the primary progenitors; (ii) disease-modifying therapies termination or the reversal of disease progression; and (iii) symptomatic treatments that treat the cognitive symptoms of the disease and protect from further cognitive decline. Among the therapeutic strategies mentioned in Figure 6, amyloid-based therapies using synthetic- as well as herbal-based antiamyloid approach are highlighted here.

### 10.1. Amyloid-Focused Therapies

Along with tau-focused therapeutic approaches, amyloid-focused treatment strategies are also in development in order to prevent the aggregation and accumulation of insoluble A*β* and/or clear A*β* plaques postformation. Still, studies have reported that soluble A*β* peptides may similarly be protective* in vivo *as an ameliorative response to free radical toxicity [116, 117].

#### 10.1.1. Inhibition of A*β* Aggregation

Since A*β* aggregation is hypothesized to be the most crucial step of the pathogenic process of AD, the strategy to inhibit A*β* aggregation has emerged as a promising approach to treat AD. Numerous synthetic as well herbal compounds have been identified as inhibitors of A*β* aggregation; however, the mechanistic interaction between A*β* and these compounds is still not clear [118]. To gain insight into the mechanism of inhibition, it is necessary to understand the structure of A*β*. While the structure of A*β* has not been resolved by crystallography, several structures have been predicted by different techniques, including nuclear magnetic resonance (NMR) spectroscopy and computer simulation [119–121]. Petkova et al. proposed a broadly used structural model for A*β* fibrils using solid state NMR (SS-NMR), as shown in Figure 5(b) [122]. In this model, residues 1–10 are structurally disordered, while residues 12–24 and 30–40 adopt *β*-strand conformations and form parallel *β*-sheets by internal hydrogen bonding. Figure 5(b) shows the secondary structure for a single A*β*_42_ monomer within the fibril. Residues 25–29 contain a 180° bend of the protein backbone that brings the two *β*-sheets in contact through side chain-side chain interactions. A single cross-*β* unit is a double-layered *β*-sheet structure with a hydrophobic core and a hydrophobic face. The only charged residues in the core are Asp23 and Lys28, which form a salt bridge to stabilize the *β*-sheet structure.

This model contains information relevant to the design of inhibitors for A*β* aggregation. For example, compounds that have interaction propensity with the hydrophobic core of A*β* peptide would disrupt monomer-monomer interaction, thereby destabilizing the formation of small oligomeric aggregates or nuclei. Compounds that recognize residues 12–24, which are included in the interaction between fibril units, would interfere with lateral association, while compounds interacting through hydrogen bonds formation with amino or carboxyl groups of residues 12–24 or 30–40 are expected to inhibit soluble aggregate elongation.

Most compounds showing inhibitory capability toward A*β* aggregation are aromatic in nature, such as resveratrol, coumarin, and nicotine [123–125]. It is hypothesized that the aromaticity plays an important role by breaking the hydrophobic interaction between A*β* monomers. Aromatic compounds can interact with residues Phe19 and Phe20 of A*β* peptide via *π*-*π* stacking interactions.

Another finding that the derivatives of penta peptide, KLVFF (residues 16–20 of A*β*), can inhibit A*β* aggregation supports this speculation [126, 127]. Therefore, to achieve better inhibitory capability toward A*β* aggregation, aromatic compounds have been modified and functionalized on their aromatic centre.

## 11. Different Model Organisms to Study Alzheimer's Disease

As the rate of occurrences of AD is growing continuously, pressure is mounting on the research community to develop a suitable and effective drug treatment. While other age-related diseases like heart disease and cancer can now be successfully studied, treated, and to a certain extent cured, AD, and other age-related human neurodegenerative diseases such as Parkinson's disease, is still not curable. This is not only due to a poorer understanding of AD, the complexity of the brain, and its relative inaccessibility, but it is also due to a lack of “natural” disease models. For example, the dog naturally mimics some AD features including A*β* cortical pathology, loss of neuronal cells, and learning and memory deficits, but it does not develop neuritic plaques and NFTs [128]. Primates do develop forms of both but are not well studied. Also, though rodents will readily develop cancer, the senile plaques and NFTs formation have never been reported [129].

Thus, transgenic AD mouse and rat models have enabled the scientific community to overcome the lack of a suitable natural model for the study of AD. The major limitation is that rodents do not naturally have anything close to AD. The AD in these models is imposed. Since the study of AD in humans is methodologically and ethically complex and critical, AD transgenic models provide an approach to understanding AD pathogenesis, so as to recognize new biomarkers and to design new therapeutics, although they do not utilize normal physiology. In addition, transgenic AD models allow investigation of the early stages of the disease, something that is problematic with human postmortem tissue [130]. In contrast numerous drug targets have “cured” these mouse models while having no benefit in clinical trials of AD patients [131]. The problem is that AD pathology is a response whose removal has limited, if any, benefit for humans but is an imposed abnormality for the transgenic rodents.

### 11.1. Transgenic Mice Model of AD

The mouse model of AD was established in the mid-1990s with the development of the PDAPP model [132], followed by the Tg2576 in subsequent years [133] and APP23 [134] models, being currently the most extensively used amyloidosis models in AD research. The PDAPP model expresses human A*β*PP carrying the Indiana familial AD mutation (V717F) driven by the platelet-derived growth factor-*β* promoter, whereas both Tg2576 and A*β*PP23 models express human A*β*PP with the Swedish mutation (K670N/M671L) driven by the hamster prion protein and murine Thy-1 promoters, respectively. All the above-mentioned models support the amyloid cascade hypothesis; they show progressive A*β* deposition in both diffuse and neuritic plaques, cerebral amyloid angiopathy, microgliosis, (limited) hippocampal atrophy, astrocytosis, synaptic and neurotransmitter alterations, and cognitive and behavioural deficits, relevant to human AD neuropathological profile [135–138]. A*β*PP-based models confirm the principal role of A*β*PP and A*β* in the Alzheimer disease process and allow target identification and subsequent preclinical evaluation of various symptomatic and disease-modifying drugs, primarily targeting the amyloid cascade. The major drawback of these models, however, is the lack of NFT formation, although hyperphosphorylated tau may be present.

The discovery of early-onset mutations in the PSEN genes aids in development of PSEN1 and PSEN2 transgenic mouse models. Even though an increased A*β*_42_/A*β*_40_ ratio in some of these models has been observed, they are void of plaque pathology. Further, few behavioural and cognitive discrepancies are present in these models; they lack NFT development like A*β*PP-based models as well. They are mainly useful for the development of double transgenic A*β*PP/PSEN mice, which display an elevated A*β*_42_/A*β*_40_ ratio and accelerated A*β* pathology compared to the single A*β*PP model they are based on, thereby supporting the modifying role of PSEN. In addition, these A*β*PP/PSEN mice display amyloid-associated inflammation, neuronal loss, cognitive decline, and BPSD-like behavioural alterations [139, 140]. The major loophole of all the above-mentioned models—lack of NFT formation—was moderately overcome by the development of transgenic mice having human tau insertion and the subsequent crossing of tau and A*β*PP models, the latter including enhanced amyloid deposition accompanied by tau hyperphosphorylation, NFT-like formation, and obvious death of neurons, thereby supporting the amyloid cascade hypothesis affirming that A*β* pathology mediates tau pathology. However, there is no colocalization of plaques and NFT in AD brain of A*β*PP/tau mice. This limitation was compensated with the development of the triple transgenic (3xTg) mouse [78]. Instead of crossing independent mutant mouse lines, two transgenic constructs (mutant A*β*PP and tau) were microinjected into single-cell embryos of homozygous mutant PSEN1 mice, thereby preventing segregation of A*β*PP and tau genes in succeeding generations. In accordance with the amyloid cascade theory, these 3xTg mice develop A*β* plaques prior to NFT pathology with a temporal and spatial profile equivalent to AD, in addition to inflammation, synaptic dysfunction, and cognitive decline [141].

The generation of transgenic rodent research models that develop some of the pathological hallmarks of AD has given a substantial boost to drug discovery efforts and has also raised many intriguing questions about the underlying disease process. However, one should never neglect the potential danger of uncritical extrapolating from mouse/rat to humans. The fact that at the moment no animal model recapitulates all aspects of human AD reflects the limitations of using a rodent system to model a human condition that takes decades to develop and primarily involves higher cognitive functions.

### 11.2. *Caenorhabditis elegans* Transgenic AD Model

*Caenorhabditis elegans*, a free-living small nematode of approximately 1.2 mm in length, has several characteristics that make it useful as a model organism. The nematodes are transparent, which allows the study of embryonic development and gene expression in living animals under the microscope. It was first used to study molecular and developmental biology by Syndey Brenner in the 1970s [142]. This invertebrate was the first animal for which an entire genome was sequenced and has become one of the most popular model organisms to study neurodegenerative disease, as demonstrated by the development of numerous transgenic disease models, including for Alzheimer's disease (discussed below).

Several AD-related genes and pathways found in humans have orthologues in* C. elegans*. The nematode genome encodes three orthologues for PSEN1: (i) sel-12, (ii) hop-1, and (iii) spe-4. The first has been found in a screen for suppressors of the egg-laying defective phenotype in lin-12 gain-of-function worms [143]; it facilitates Notch/lin-12 signalling, functional mostly during embryonic development. The second, that is hop-1, homolog of PSEN1 [144], in fact shares more homology to human PSEN2; and the third, spe-4, has no clear human counterpart [144]. Three genes, aph-1, pen-2, and aph-2, combine together to form a functional complex of *γ*-secretase. In addition, an orthologue of A*β* (apl-1) has been described in* C. elegans* [145]. Similar to* Drosophila*, the APL-1 protein does not contain the A*β* sequence; neither does* C. elegans* display BACE1-like activity.

There are basically three A*β*-expressing nematode models which have been developed. When expressed in muscle cells, A*β*_1–42_ induced the formation of amyloid-immunoreactive inclusions. A subset of these deposits also binds the A*β*-specific dye thioflavin S, showing that amyloid fibrils are formed similar to human AD. In addition, paralysis of the nematodes occurred, thereby indicating a muscle cell specific toxicity of A*β* [146]. Nematodes expressing A*β*_1–42_ in neuronal cells also develop A*β* deposits but display only a very subtle phenotype [147]. Interestingly, oligomeric A*β* were detected in these strains that might be similar to the neurotoxic A*β*-derived diffusible ligands [148]. These transgenic models provide important insight into the toxicity of specific A*β* species but do not allow screening of chemical or genetic modifiers of A*β*PP processing.

To create tauopathy models of nematode, both wild-type and mutated human tau proteins were expressed in neurons of* C. elegans*, inducing a progressive phenotype of defective motility (uncoordinated phenotype), which was more deceptive in the mutants. Interestingly, these transgenic lines also display hyperphosphorylation of tau [149], which is linked to GSK-3*β* activation. Future genome-wide screens will display which modifier genes are linked to the complex disease process and characterize diagnostic or therapeutic drug targets.

### 11.3. *Drosophila melanogaster* as a Transgenic Model of AD

*Drosophila melanogaster* are the most commonly used species of* Drosophila* in the laboratory worldwide for research purposes. Their use in the modelling of human neurodegenerative disease is based on the inherent assumption that the fundamental aspects of cell biology are conserved throughout evolution in higher organisms. This is supported by the fact that ~75% of human disease-related genes have homologs in* Drosophila*, suggesting that molecular mechanisms of any disease in humans may be conserved in the fly.

There are many compelling reasons to study AD in the* Drosophila* model. The* Drosophila* brain has approximate 300,000 neurons and is organized into the area with separate, specialized functions such as memory, learning, olfaction, and vision, similar to human.* Drosophila*, on account of its very short generation time (10–12 days) and easy maintenance, is a popular model in genetic research. Although one could argue that the* Drosophila*'s maximum lifespan of 55–80 days is significantly greater than that of the worm (~18 days), it is still much shorter than that of the mouse (2-3 years), making it ideal for studying a progressive age-related disease such as AD.

In addition, the* Drosophila* has an unrivalled battery of genetic tools, including a fully sequenced genome; an extensive library of mutant stocks including RNA interference (RNAi) and knockout (KO) lines; sophisticated transposon-based methods for gene manipulation; systems for spatial and temporal specific ectopic gene expression; and balancer chromosomes. Balancer chromosomes are unique: composed of multiple inversions that prevent recombination, together with dominant, lethal, and visible markers. They allow the maintenance in long-term culture of lethal or deleterious mutations in heterozygotes, without the necessity to set up specific crosses.

The combination of such extensive genetic tools and practicality makes the* Drosophila* ideal for genetic screening. A variety of screening methods are available in the* Drosophila*, involving chemical mutagenesis (EMS), genetic deletion kits, or mobile genetic elements (P, EP, and GS elements). Genetic screens are powerful experiments providing an unbiased forward genetic approach, which allows the discovery of genes or metabolic pathways not immediately apparent in the pathogenesis of AD.

### 11.4. Drug Screens Using* Drosophila* AD Models

Another potential use for a characterized* Drosophila* disease model is its use for novel drug screening. The secreted A*β* peptide fly model was verified as a platform for drug discovery by testing the efficacy of a drug used to treat human AD patients and was shown to slow progression of AD [150]. The drug memantine, a noncompetitive glutamate antagonist, is effective in slowing progression of human AD [151]. In addition, the life span of flies expressing two copies of A*β*_42_ or one copy of A*β*_42_-arctic was increased when flies were treated with MK-801, an inhibitor of the excitatory action of glutamate on the NMDA receptor [150]. A therapeutic intervention that is effective in human AD patients is, therefore, also effective in the fly; thus the fly AD model is useful for testing novel human drugs. Congo red, which binds to A*β* and has been shown to reduce neurodegeneration in a fly model of polyQ disease [152] and a mouse model of Huntington's disease [153], has also been shown to reverse the reduced life span of flies expressing two copies of A*β*_42_ or one copy of A*β*_42_-arctic [150].

The A*β*PP processing model in* Drosophila* has also been used in drug validation studies. Ubiquitous expression of A*β*PP, BACE, and DPsn resulted in reduced longevity and a visible wing phenotype, which were used for screening *β*- and *γ*-secretase inhibitors [154]. Feeding flies with either *β*- or *γ*-secretase inhibitors resulted in an increased survival of A*β*PP/BACE/DPSn expressing transgenic flies, making this fly model useful for investigating drugs that modulate A*β*PP processing and have the potential to decrease A*β*-induced cellular degeneration [154]. Singh in his doctoral thesis showed neuroprotective effect of some herbal compounds targeting anti-A*β* therapeutic approach using* Drosophila* model of AD [155].

## 12. A*β* and Metallosis

### 12.1. Metal Ions and A*β* Toxicity

In case of AD, elevated metal ions concentrations have been demonstrated in several studies [156–159], in particular copper and zinc which are associated with both the aggregation and the neurotoxicity of A*β* peptides, and proposed as an important factor in neuropathology of AD [156, 159–162].

**Figure 7:**
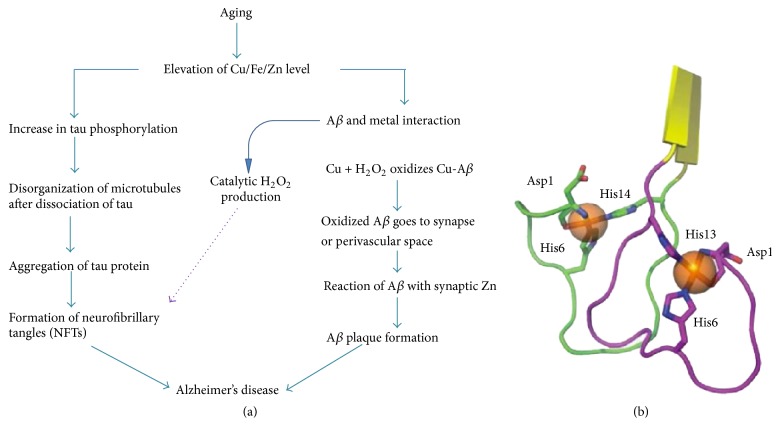
(a) Proposed model for AD pathology based on abnormal metal interaction. Cu and Fe levels increase during aging in the CNS and result in increase in metal and A*β* interaction. Cu binding to A*β* results in ROS production and autooxidation of A*β* peptide. Oxidized A*β* contributes to synaptic pathology and plaque formation. Metals may also promote phosphorylation of tau and hence enhance formation of NFT which further contribute to AD pathology. (b) Model showing the amyloid-copper interaction. Notice the coordination sites at His 6 from one peptide together with His13 and His14 from the second peptide.

An extensive number of reports have provided empirical data showing metal mediated toxicity of amyloid-*β*, but a detailed NMR or X-ray diffraction atomic structure is yet to be described [163, 164]. However, Azimi and Rauk [165] were able to use MD simulations to demonstrate that A*β*-copper coordinated structures can form both parallel and antiparallel conformations. Zinc ions have been shown to form intermolecular complexes while copper ions tend to form intramolecular complexes cross-linking multiple peptides [166–168]. The schematic in Figure 7(a) shows the details of metal ion mediated A*β* toxicity which results in fibril formation and leads to AD.  Figure 7(b) depicts A*β*-copper interactions [162]. Interaction of A*β* amino acid residues with copper is shown in figure which leads to Cu mediated A*β* toxicity. Copper has been shown to interact with amyloid-*β* at the His13 and His14 residues on one peptide with the His6 residue on the other peptide.

Histidine is well known as Zn^2+^ and Cu^2+^ ligand for many other proteins and peptides [169, 170]. Coordination of Zn^2+^ to His13 and His14 but not His6 has been found to be critical to induce A*β*_1–40_ aggregation [171, 172]. Cu^2+^ is able to compete with Zn^2+^ for binding to histidine residues of A*β* and at low concentration it inhibits the ability of Zn^2+^ to induce aggregation, but at higher Cu^2+^ concentrations aggregation does occur [173].

It has been reported that the levels of copper (0.4 ± 0.1 mg/g of wet weight of plaque) and zinc (1.2 ± 0.2 mg/g) are found to be high in the senile plaques found within AD brains [157]. Use of a Cu-Zn chelator, such as clioquinol, inhibits A*β* accumulation in AD transgenic mice [174, 175], which highlights the importance of studying copper and zinc binding to A*β*.

### 12.2. ROS Generation by Metal Mediated A*β* Toxicity

The coordination of metal ions such as copper, iron, and zinc to A*β* also results in the chemical reduction of these metals and the subsequent generation of hydrogen peroxide from molecular oxygen together with other available biological reducing agents such as cholesterol, in a catalytic manner [176–178]. In the case of A*β*_42_, the reduction of copper is independent of the aggregation state of the peptide, as both soluble and fibrillar forms show copper-reducing ability.

The generation of hydrogen peroxide in the presence of reduced metals, and in the absence of sufficient detoxifying enzymes such as catalase and glutathione peroxidase, gives rise to the toxic hydroxyl radical via Fenton chemistry [176]. The generation of hydrogen peroxide contributes to A*β* toxicity. In support of this, cellular toxicity can be rescued by the addition of catalase [178, 179]. Further, resistance to A*β* toxicity is associated with an enhanced ability to degrade hydrogen peroxide [180], and catalase inhibitors can enhance A*β* toxicity. The potentiation of A*β* toxicity by copper is the greatest for A*β*_42_ > A*β*_40_ > rodent A*β*_40_, which corresponds to the peptide relative activities in reducing copper(II) to copper(I) [176]. These data support a role for A*β* in the generation of hydrogen peroxide via metal ion reduction and for oxidative processes in the augmentation of A*β* to potentiate the AD cascade. A summary of the proposed role of metal ions in AD has already been shown in Figure 7(a).

### 12.3. Neuroprotective Role of Metal Chelator against A*β* Induced Toxicity

To prevent metal mediated neurotoxicity of A*β*, researchers are focusing on chelation therapy. Chelation therapy is the use of metal specific chelators which are able to chelate extra metal ions present in brain; hence reduced possibility of interaction of these metals with A*β*, and ultimately a slowdown of metal, mediated A*β*-toxicity.

Traditional metal chelators have been used to sequester or redistribute metal ions from metal-bound A*β* species in order to suppress metal mediated A*β* neurotoxicity* in vitro* and* in vivo* [181–184]. Cherny et al. [174] initially reported that Cu/Zn chelators solubilize A*β* from tissues of postmortem AD brain. They choose the clioquinol (CQ, 5-chloro-7-iodo-8-hydroxyquinoline) based on its ability to cross the blood-brain barrier as tested in AD transgenic mice (Tg2576) and found significantly reduced levels (~65%) of A*β*-aggregates as well as ROS generation in CQ treated mice as compared to control.

Ongoing research in this area focuses on the prevention of metal mediated A*β* neurotoxicity and ROS production by metal chelating therapy, which is an emerging trend in current research. There is immense need to develop such a suitable metal chelator that can prevent A*β* aggregation by effectively sequestering extra metal ions. Several groups focused on developing such type of new molecules [182, 185, 186]. More particularly, in a pioneering work, Lakatos et al. developed two carbohydrate-containing compounds, N,N′-bis[(5-*β*-D-glucopyranosyloxy-2-hydroxy)benzyl]-N,N′-dimethyl-ethane-1,2-diamine (H2GL1) and N,N′-bis[(5-*β*-D-glucopyranosyloxy-3-tert-butyl-2-hydroxy)benzyl]-N,N′-dimethyl-ethane-1,2-diamine (H2GL2), that are shown to be promising therapeutic tools against AD, based on* in vitro* studies [185]. In this context, we designed and synthesized novel compound L, 2,6-pyridinedicarboxylic acid, 2,6-bis[2-[(4-carboxyphenyl)methylene]hydrazide], to test the* in vivo* neuroprotective efficacy in a well-established* Drosophila* transgenic model system. Recently Singh et al. reported the neuroprotective role of a novel copper chelator against copper mediated A*β* toxicity [162].

## 13. Neuroprotective Role of Flavonoid against AD

Naturally occurring as well as synthetically synthesized dietary flavonoids have been extensively used as alternative candidates for Alzheimer's treatment, taking into account their antioxidative, antiamyloidogenic, and anti-inflammatory properties. Experimental evidence from different studies supports the hypothesis that certain flavonoids may protect against AD, in part by interfering with the generation and assembly of amyloid-*β* peptides into neurotoxic oligomeric aggregates and also by reducing tau aggregation. Dietary supplementation studies using flavonoid-rich plant or food extracts have shown their ability to influence cognition and learning in humans and also in animal models of diseases [187–192]. Presently, there is no direct association between flavonoid consumption and improvement in neurological health. Nevertheless, the potential beneficial effect of flavonoids in the brain seems to be related to their ability to interact with intracellular neuronal and glial signalling pathways, thus influencing the peripheral and cerebral vascular system, protecting vulnerable neurons, enhancing existing neuronal function, or stimulating neuronal regeneration.

Flavonoids are naturally occurring polyphenolic compounds widely spread in plants. They are present in foods and beverages of plant origin such as a variety of fruits, vegetables, cocoa, cereals, tea, and wine [193]. The six main subclasses of flavonoids include (1) flavonols (e.g., kaempferol, quercetin), present in onions, leeks, and broccoli; (2) isoflavones (e.g., daidzein, genistein), found mainly in soy and soy products; (3) flavones (e.g., apigenin, luteolin), present in parsley and celery; (4) flavanols (e.g., catechin, epicatechin, epigallocatechin, and epigallocatechin gallate (EGCG)), abundant in green tea, red wine, and chocolate; (5) flavanones (e.g., hesperetin, naringenin), primarily found in citrus fruit and tomatoes; and finally (6) anthocyanidins (e.g., pelargonidin, cyanidin, and malvidin), sources of which include berry fruits and red wine.

It was thought that the ability of flavonoids to promote memory, learning, and cognitive function was mediated by their antioxidant capacity [194]. Nevertheless, due to their limited absorption and their low bioavailability in the brain, increasing evidence demonstrates that they are able to interact with the cellular and molecular components of the brain responsible for memory, having the potential to protect vulnerable neurons, enhance existing neuronal function, stimulate neuronal regeneration, and induce neurogenesis [194, 195]. Recent study from our lab showed the neuroprotective property of a novel synthetic flavonoid derivative against A*β*-induce neurotoxicity in* Drosophila* model of AD [196].

## 14. Neuroprotective Role of Natural Polyphenols in AD

Nature has gifted mankind with a plethora of vegetables, flora-bearing fruits, and nuts. Natural polyphenols are the most commonly found chemical compounds in consumable herbal beverages and food worldwide [197, 198]. They constitute a large group of phytochemicals with more than 8000 identified compounds. The variety of bioactive nutrients present in these natural products play a central role in prevention and cure of various human neurodegenerative diseases, such as Alzheimer's disease (AD), Parkinson's disease, and other kinds of neuronal damage. Plants have a long history as a rich source of new bioactive compounds for drug discovery and may have advantages in relation to efficacy. Several reports documented the effectiveness of herbal extracts over isolated material, in protection against lipid peroxidation [199], and anticancer effects [200]. For example, a mixture of carotenoids has been found to be more effective than any one single carotenoid in protecting liposomes against lipid peroxidation [199].

Polyphenolic compounds from medicinal plants are key sources of neuroprotective agents against AD. Using the structure of these bioactive ingredients as templates for synthetic drugs offers a wide range of potential neuroprotective compounds [201]. In the past few decades, several studies attempted to measure the effect of total plant extract on AD and to isolate the active component responsible for the neuroprotective effects [202, 203].

Natural polyphenols reveal their antioxidant effect by reducing free radical species and/or encouraging endogenous antioxidant capacity. Thus, the antioxidant properties positively contribute to their neuroprotective effects. Furthermore, some of them influence synthesis of endogenous antioxidant molecules in cells via activating Nrf/ARE pathway [204]. Apart from antioxidant property, most of them appear to have a number of different molecular targets, affecting several signalling pathways and showing pleiotropic activity on cells [205]. For instance, polyphenolic compounds can modulate activity of NF-*κ*B or SIRT1 exerting neuroprotective effects. Recent studies have shown anti-A*β* activity of compounds from natural sources* in vitro* and* in vivo* [206–208]. Still, evidence for the capability of common edible elements to inhibit A*β* oligomerization* in vivo* remains a challenge.

**Figure 8:**
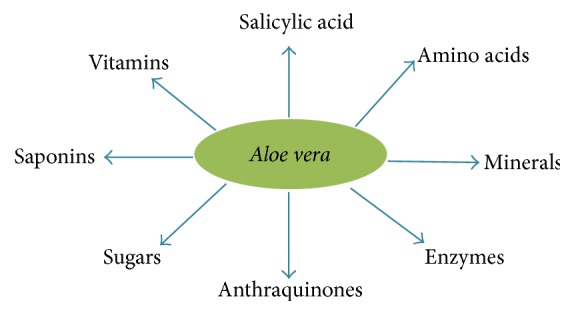
Illustration of the different components present in* Aloe vera*.

**Figure 9:**
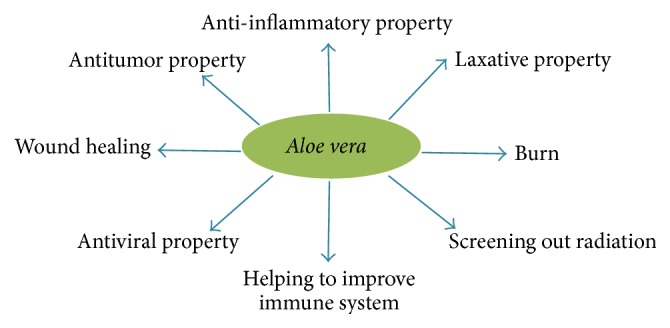
Figure showing the medicinal properties of* Aloe vera*.

*Aloe vera* has been used as medicinal agent since Roman times [209].* A. vera* contains different bioactive components (Figure 8) harbouring over 75 biologically active compounds [210] known to have a wide range of pharmacological activities (Figure 9), including anti-inflammatory, wound healing, antioxidative, antiarthritic, antidiabetic, and antitumorigenic effects [211].

*Aloe vera* has always been preferred as a herbal remedy and is one of the most popular herbal plants. Major value added products from* Aloe* are gel and juice.

Recently, it has been reported that* Aloe vera*, supplemented orally to mice, is effective on wound healing.* Aloe vera* acts as a free radical scavenger and has other antioxidant properties on diabetic patients by controlling elevated anions in an alloxan- or STZ-induced diabetic animal models [212, 213].

## 15. Conclusion

Currently the accumulated experimental evidence leans toward strongly supporting the toxic role of A*β* within the pathophysiology of AD. However, the existence of some data regarding the role of A*β* in the normal physiology of the brain suggests that this peptide may act in different modes at different times, according to diverse conditions. So far, it appears that at the initial stages of development and in the young brain, when in physiological doses (i.e., picomolar to nanomolar range) and in soluble, oligomeric forms, A*β* can show neuroprotective, antioxidant, and trophic properties, even facilitating synaptic plasticity. On the contrary, in many potentially adverse conditions, A*β* may deploy multiple toxic effects, contributing significantly to neuronal damage, as seen in AD. Some of these conditions appear to be associated with A*β* itself, such as high concentrations and fibrillar or aggregated states, presence of free metals, brain tissue previously injured or aged, and decreased antioxidative mechanisms. Moreover, it is necessary to remark that both trophic and toxic effects may not be mutually exclusive. In other words, they might coexist and cross-modulate each other, even throughout advanced stages of AD, complicating an approach based upon antiamyloidogenic therapy, at least theoretically. This functional duality may also underlie the modest success and the high rate of collateral consequences of such therapies. In summary, blockade, inhibition, or modulation of those sites, effects, and negative processes in which A*β* is involved, but simultaneously respecting those sites and physiologic processes in which A*β* is also taking part, remain a major challenge for therapeutic research in the future.
